# Outcomes of Distal Pancreatectomy for Pancreatic Ductal Adenocarcinoma in the Netherlands: A Nationwide Retrospective Analysis

**DOI:** 10.1245/s10434-015-4930-4

**Published:** 2015-10-27

**Authors:** Thijs de Rooij, Johanna A. Tol, Casper H. van Eijck, Djamila Boerma, Bert A. Bonsing, Koop Bosscha, Ronald M. van Dam, Marcel G. Dijkgraaf, Michael F. Gerhards, Harry van Goor, Erwin van der Harst, Ignace H. de Hingh, Geert Kazemier, Joost M. Klaase, I. Quintus Molenaar, Gijs A. Patijn, Hjalmar C. van Santvoort, Joris J. Scheepers, George P. van der Schelling, Egbert Sieders, Olivier R. Busch, Marc G. Besselink, for the Dutch Pancreatic Cancer Group

**Affiliations:** Department of Surgery, Academic Medical Center, Amsterdam, The Netherlands; Department of Surgery, Erasmus Medical Center, Rotterdam, The Netherlands; Department of Surgery, St Antonius Hospital, Nieuwegein, The Netherlands; Department of Surgery, Leiden University Medical Center, Leiden, The Netherlands; Department of Surgery, Jeroen Bosch Hospital, Den Bosch, The Netherlands; Department of Surgery, Maastricht University Medical Center, Maastricht, The Netherlands; Clinical Research Unit, Academic Medical Center, Amsterdam, The Netherlands; Department of Surgery, Onze Lieve Vrouwe Gasthuis, Amsterdam, The Netherlands; Department of Surgery, Radboud University Medical Center, Nijmegen, The Netherlands; Department of Surgery, Maasstad Hospital, Rotterdam, The Netherlands; Department of Surgery, Catharina Hospital, Eindhoven, The Netherlands; Department of Surgery, VU Medical Center, Amsterdam, The Netherlands; Department of Surgery, Medisch Spectrum Twente, Enschede, The Netherlands; Department of Surgery, University Medical Center Utrecht, Utrecht, The Netherlands; Department of Surgery, Isala Clinics, Zwolle, The Netherlands; Department of Surgery, Reinier de Graaf Gasthuis, Delft, The Netherlands; Department of Surgery, Amphia Hospital, Breda, The Netherlands; Department of Surgery, University Medical Center Groningen, Groningen, The Netherlands

## Abstract

**Background:**

Large multicenter series on outcomes and predictors of survival after distal pancreatectomy (DP) for pancreatic ductal adenocarcinoma (PDAC) are scarce.

**Methods:**

Adults who underwent DP for PDAC in 17 Dutch pancreatic centers between January 2005 and September 2013 were analyzed retrospectively. The primary outcome was survival, and predictors of survival were identified using Cox regression analysis.

**Results:**

In total, 761 consecutive patients after DP were assessed, of whom 620 patients were excluded because of non-PDAC histopathology (*n* = 616) or a lack of data (*n* = 4), leaving a total of 141 patients included in the study [45 % (*n* = 63) male, mean age 64 years (SD = 10)]. Multivisceral resection was performed in 43 patients (30 %) and laparoscopic resection was performed in 7 patients (5 %). A major complication (Clavien–Dindo score of III or higher) occurred in 46 patients (33 %). Mean tumor size was 44 mm (SD 23), and histopathological examination showed 70 R0 resections (50 %), while 30-day and 90-day mortality was 3 and 6 %, respectively. Overall, 63 patients (45 %) received adjuvant chemotherapy. Median survival was 17 months [interquartile range (IQR) 13–21], with a median follow-up of 17 months (IQR 8–29). Cumulative survival at 1, 3 and 5 years was 64, 29, and 22 %, respectively. Independent predictors of worse postoperative survival were R1/R2 resection [hazard ratio (HR) 1.6, 95 % confidence interval (CI) 1.1–2.4], pT3/pT4 stage (HR 1.9, 95 % CI 1.3–2.9), a major complication (HR 1.7, 95 % CI 1.1–2.5), and not receiving adjuvant chemotherapy (HR 1.5, 95 % CI 1.0–2.3).

**Conclusion:**

Survival after DP for PDAC is poor and is related to resection margin, tumor stage, surgical complications, and adjuvant chemotherapy. Further studies should assess to what extent prevention of surgical complications and more extensive use of adjuvant chemotherapy can improve survival.

Pancreatic ductal adenocarcinoma (PDAC) is the fifth leading cause of cancer death and is associated with a dismal 5-year cumulative survival of 6 %.^1^ PDAC is one of the most aggressive cancers and, to date, only surgery, in combination with adjuvant chemotherapy, has the potential to achieve long-term survival. Unfortunately, only a minority (15–20 %) of patients are eligible for surgical resection at the time of diagnosis, mainly because of the late onset of nonspecific symptoms, resulting in the frequent presence of metastatic or locally advanced disease.^2^–^4^ Of the two main surgical procedures for PDAC, distal pancreatectomy (DP) has traditionally been considered less complicated (i.e. less complications, lower mortality) than pancreatoduodenectomy. Merely one-fifth of all pancreatic cancers is located in the pancreatic tail and, of these patients, 80 % have unresectable disease due to metastasis or major vessel tumor invasion.^4^–^7^

Of the patients who can undergo DP for PDAC, reports on survival in large cohorts are scarce, as opposed to pancreatoduodenectomy.^8^,^9^ This may be explained by the low volume characteristics of DP for PDAC. To date, two multicenter series on survival after DP for PDAC have been published. The first series from the US found a median postoperative survival of 16 months, whereas a French study reported a median postoperative survival of 35 months.^10^,^11^ These series also assessed independent predictors of survival, but again with conflicting outcomes. Therefore, it is not yet clear how outcomes in these patients can potentially be improved. This multicenter study was designed to assess overall survival and predictors of survival after DP for PDAC.

## Methods

### Patients and Design

This was a nationwide retrospective study including all consecutive adult patients who had undergone DP for PDAC in 1 of 17 pancreatic centers of the Dutch Pancreatic Cancer Group (DPCG) between 1 January 2005 and 1 September 2013. Each of these centers performs at least 20 pancreatoduodenectomies annually, as of 2007. Patients were excluded when postoperative histopathological diagnosis was not PDAC or when essential data on the surgical procedure or pathology report were lacking. This study was conducted according to the Strengthening the Reporting of Observational studies in Epidemiology (STROBE) statement.^12^ Data were registered anonymously and evaluated retrospectively, without burden for the patient. Therefore, according to the local Medical Ethics Review Committee, informed consent was not required. Socioeconomic status was not considered to have any influence on access of care in this series as in The Netherlands every citizen is covered by a health insurance policy.

### Surgical Technique and Oncologic Treatment Regimen

Preoperative chemotherapy or radiotherapy was not used within the current timeframe. DP was performed via an open or laparoscopic approach. In case of proven or suspected malignant disease, a subsequent splenectomy was generally performed. Routine lymphadenectomy was performed according to the International Study Group on Pancreatic Surgery (ISGPS) recommendations, and also before the ISGPS publication regarding standard lymphadenectomy.^13^ The pancreas was transected using either sharp transection or a stapling device. Additional sutures or an absorbable fibrin sealant patch were used in a small subset of patients at the discretion of the surgeon. In all patients, a surgical drain was placed near the pancreatic remnant and left subphrenic space. Patients received adjuvant chemotherapy when they were fit enough (according to the medical oncologist), and they chose to receive it. As of 2011, the Dutch guideline on pancreatic cancer advised the use of adjuvant gemcitabine after resection for PDAC.^14^

### Definitions

Multivisceral resection was defined as resection of any organ or a part of an organ besides the pancreas, spleen, and adrenal gland. Resection margins, including transection and circumferential margins, were classified as R0 (distance margin to tumor ≥1 mm), R1 (distance margin to tumor <1 mm), or R2 (macroscopically positive margin).^15^ PDAC stages were classified according to the American Joint Committee on Cancer (AJCC; 7th edition) staging by pT, pN, and pM stages, and overall cancer stages 1A, 1B, 2A, 2B, III, and IV.^16^ Postoperative complications were scored using the Clavien–Dindo classification of surgical complications,^17^ and major complications were defined as complications with a Clavien–Dindo score of III or higher. Additionally, specific complications such as grade B/C postoperative pancreatic fistula, delayed gastric emptying, and postpancreatectomy hemorrhage were all scored using the recommended ISGPS definitions.^18^–^20^ Surgical site infection was defined using the Centers for Disease Control and Prevention (CDC) definition.^21^

### Outcomes and Data Collection

Data were collected from patient records and patient charts with daily notes. Baseline parameters collected included sex, age, body mass index, American Society of Anesthesiologists (ASA) physical status, history of abdominal surgery, and preoperative signs of ingrowth in adjacent organs. The primary study outcome was survival. Secondary outcomes were intraoperative and postoperative parameters, such as splenectomy, multivisceral resection, procedure time, intraoperative blood loss, resection margins, lymph node retrieval, postoperative pancreatic fistula, delayed gastric emptying, postpancreatectomy hemorrhage, surgical site infection, intensive care unit admission, length of stay, readmission within 30 days, adjuvant chemotherapy, 30-day mortality, 90-day mortality, and 1-, 3-, and 5-year cumulative survival. Complications were collected up to 90 days postoperatively. Clavien–Dindo morbidity scores were registered when they were recorded by the hospital where the patient was operated, while mortality rates were used to display all deaths within a mentioned timeframe. Survival data were collected in February 2015 for all patients using the municipal personal records database, a registry that contains the personal details of every Dutch inhabitant.

### Statistical Analysis

Data were analyzed using IBM SPSS^®^ version 20.0 (IBM Corporation, Armonk, NY, USA). Dichotomous data were expressed as proportions, and continuous data were expressed as means and standard deviations (SDs) or medians and interquartile ranges (IQRs), depending on their distribution. Dichotomous data were compared using a *χ*^2^ analysis, while continuous data were compared using an independent Student’s *t* test or Mann–Whitney *U* test as appropriate. Survival was assessed using Kaplan–Meier analysis, from the date of DP until death or last moment of follow-up (censored observation). Comparison of subgroups (i.e. patients who received chemotherapy vs. patients who did not receive chemotherapy, and patients who underwent DP only vs. patients who underwent multivisceral resection) with regard to survival was performed using a log-rank test. Univariable and multivariable Cox regression analyses with backward selection were performed to identify predictors of survival. Parameters with a *p* value < 0.1 in the univariable analysis were included in the multivariable analysis. Results of the univariable and multivariable analyses were expressed as hazard ratios (HRs) with corresponding 95 % confidence intervals (CIs). A subgroup analysis comparing patients who received adjuvant chemotherapy with patients who did not receive adjuvant chemotherapy was performed. A two-tailed *p* value < 0.05 was considered statistically significant.

## Results

### Patient Characteristics

In total, 761 patients had undergone DP within the study period, as described previously.^22^ For this study, 620 patients were excluded: 616 patients because the histopathological diagnosis was other than PDAC, and 4 patients because data were lacking. Therefore, 141 patients who had undergone DP for PDAC were included in this study (Table 1). The median annual volume increased from seven procedures in the first 3 years of the study period to 25 procedures in the last 3 years.

**Table 1:** Baseline characteristics of 141 patients undergoing distal pancreatectomy for pancreatic ductal adenocarcinoma

Male sex	63 (45)
Age [years; mean (SD)]	64 (10)
BMI [kg/m^2^; mean (SD)]	25 (4)
ASA physical status	
1	16 (11)
2	99 (70)
3	23 (16)
4	3 (2)
Previous abdominal surgery	53 (38)
Other organs involved on CT/MRI	29 (21)
Stomach	9 (6)
Spleen	7 (5)
Kidney	4 (3)
Intestine	4 (3)
Two or more adjacent organs	5 (4)

Data are expressed as *n* (%) unless otherwise specified

*SD* standard deviation, *BMI* body mass index, *ASA* American Society of Anesthesiologists, *CT* computed tomography, *MRI* magnetic resonance imaging

### Intraoperative and Pathology Outcomes

DP with splenectomy was performed in 124 patients (88 %) and multivisceral resection was performed in 43 patients (30 %), as shown in Table 2. Laparoscopic resection was performed in seven patients (5 %). Median procedure time was 194 min (IQR 150–270), and median intraoperative blood loss was 800 mL (IQR 495–1618). An absorbable fibrin sealant patch was used in 17 patients (12 %). Pathology reports demonstrated a mean tumor size of 44 mm (SD 23). Microscopically radical transection and circumferential resection margins (R0 resection) were obtained in 70 patients (50 %), of whom 17 patients had undergone multivisceral resection. The median number of resected lymph nodes, as shown in Table 2, was not influenced by hospital volume or the patient’s body mass index.

**Table 2:** Operative and pathology outcomes of 141 patients undergoing distal pancreatectomy for pancreatic ductal adenocarcinoma

Splenectomy	124 (88)
Multivisceral resection	43 (30)
Stomach	9 (6)
Intestine	5 (4)
Kidney/adrenal gland	5 (4)
Vessel resection	5 (4)
Other	4 (3)
>1 additional resection	15 (11)
Procedure time [min; median (IQR)]	194 (150–270)
Blood loss [min; median (IQR)]	800 (495–1618)
Tumor size [mm; mean (SD)]	44 (23)
Resection margins	
R0	70 (50)
R0 in multivisceral resection	17 (40)
R1	64 (45)
R2	7 (5)
Lymph nodes resected [median (IQR)]	8 (4–14)
Positive lymph nodes resected [mean (SD)]	1 (1)
pT stage	
1	14 (10)
2	70 (50)
3	52 (37)
4	5 (4)
pN1 stage	74 (53)
pM1 stage	4 (3)
Cancer stage	
1A	6 (4)
1B	34 (24)
2A	25 (18)
2B	68 (48)
3	4 (3)
4	4 (3)

Data are expressed as *n* (%) unless otherwise specified

*IQR* interquartile range, *SD* standard deviation

### Postoperative Outcomes

Details of the postoperative clinical course are shown in Table 3. Postoperatively, 33 % of patients (*n* = 46) developed a major complication. A postoperative pancreatic fistula grade B/C occurred in 24 patients (17 %). Median length of stay was 10 days (IQR 8–15). Postoperative 30-day and 90-day mortality were 3 % (*n* = 4) and 6 % (*n* = 9), respectively. Three patients (2 %) died within 90 days postoperatively for unknown reasons, which was not recorded by the hospital where these patients were operated. In total, 63 patients (45 %) received adjuvant chemotherapy.

**Table 3:** Postoperative outcomes of 141 patients undergoing distal pancreatectomy for pancreatic ductal adenocarcinoma

Major complications	46 (33)
Clavien–Dindo 3	35 (25)
Clavien–Dindo 4	5 (4)
Clavien–Dindo 5	6 (4)
Postoperative pancreatic fistula grade B/C	24 (17)
Delayed gastric emptying grade B/C	25 (18)
Postpancreatectomy hemorrhage grade B/C	6 (4)
Surgical site infection	18 (13)
Intensive care unit admission	24 (17)
Length of intensive care unit stay [days; median (IQR)]	2 (2–8)
Length of stay [days; median (IQR)]	10 (8–15)
Readmission within 30 days	25 (18)
Adjuvant chemotherapy	63 (45)
Mortality ≤30 days postoperatively	4 (3)
Mortality ≤90 days postoperatively	9 (6)

Data are expressed as *n* (%) unless otherwise specified

*IQR* interquartile range

### Survival

Median follow-up was 17 months (IQR 8–29), and no patients were lost to follow-up for survival. In this cohort, 100 patients died and 41 patients were censored for survival analysis. Postoperative median survival was 17 months (95 % CI 13–21), while 1-, 3-, and 5-year cumulative survival were 64, 29, and 22 % respectively. Survival did not differ between patients who did and did not undergo splenectomy. Survival was worse in patients who had undergone DP with multivisceral resection [10 months (95 % CI 6–15) vs. 22 months (95 % CI 17–27); *p* < 0.01]. In the univariable analyses, multivisceral resection, R1/R2 resection, pT3 stage, a major complication, and not receiving adjuvant chemotherapy were associated with worse survival.

In the multivariable analysis, R1/R2 resection, pT3/pT4 stage, a major complication, and not receiving adjuvant chemotherapy were significant predictors of worse survival (Table 4). Adding sex and age to the multivariable Cox regression analysis did not change the statistical significance of the included variables.

**Table 4:** Univariable and multivariable Cox regression analyses for predictors of survival after distal pancreatectomy for pancreatic ductal adenocarcinoma

Variable	Univariable analysis		Multivariable analysis	
	HR (95 % CI)	*p* value	HR (95 % CI)	*p* value
Female sex	0.88 (0.59–1.31)	0.52	–	–
Age > 70 years	1.30 (0.85–1.99)	0.22	–	–
ASA physical status 3/4	1.06 (0.64–1.77)	0.82	–	–
Tumor size (cm)	1.03 (0.96–1.11)	0.39	–	–
Laparoscopic approach	1.31 (0.57–3.00)	0.52	–	–
Multivisceral resection	2.04 (1.35–3.09)	<0.01	–^a^	–^a^
R1/R2 resection	1.81 (1.22–2.70)	<0.01	1.61 (1.08–2.41)	0.02
No. of tumor-positive lymph nodes	1.06 (0.94–1.18)	0.35	–	–
pN1 stage	1.41 (0.95–2.10)	0.09	–^a^	–^a^
pT3/pT4 stage	3.84 (1.71–8.60)	<0.01	1.92 (1.29–2.88)	<0.01
Major complication	1.74 (1.16–2.62)	0.01	1.67 (1.10–2.51)	0.02
No adjuvant therapy	1.89 (1.23–2.90)	<0.01	1.51 (1.01–2.26)	0.045

*ASA* American Society of Anesthesiologists, *HR* hazard ratio, *CI* confidence interval

^a^ Included in the multivariable analysis but removed during backward selection

### Subgroup Analyses

Patients who received adjuvant chemotherapy (*n* = 63) did not differ from those patients who did not receive adjuvant chemotherapy (*n* = 78) with regard to male sex [34 (54 %) patients vs. 44 (56 %) patients; *p* = 0.77], mean age [63 (SD 11) vs. 65 (SD 9) years; *p* = 0.43], ASA physical status 1/2 [51 (81 %) patients vs. 64 (82 %) patients; *p* = 0.87], tumor stage [pT1, 9 (14 %) vs. 5 (6 %) patients; pT2, 33 (52 %) vs. 37 (47 %) patients; pT3, 19 (30 %) vs. 33 (52 %) patients; and pT4, 2 (3 %) vs. 3 (4 %) patients; *p* = 0.29], the presence of lymph node metastases [33 (52 %) patients vs. 41 (53 %) patients; *p* = 0.98], or tumor size (43 vs. 44 mm; *p* = 0.68). Patients with a major complication (*n* = 46) were less likely to receive adjuvant chemotherapy compared with patients without a major complication (*n* = 95) [14 (30 %) patients vs. 49 (51 %) patients; *p* = 0.02]. Furthermore, a major complication was a predictor of not receiving adjuvant chemotherapy in a multivariable logistic regression analysis (odds ratio 2.4, 95 % CI 1.2–1.3; *p* = 0.02), in which sex, age, ASA physical status, and a major complication were included (a laparoscopic approach had a *p* value < 0.2 in a univariable analysis and was therefore not included in the multivariable analysis). After publication of the Dutch guideline on pancreatic cancer in 2011, more patients received adjuvant chemotherapy [30/48 (63 %) vs. 33/93 (35 %); *p* < 0.01]. Patients who received adjuvant chemotherapy had a significantly better median survival than patients who did not receive adjuvant chemotherapy [23 months (95 % CI 17–29) vs. 12 months (95 % CI 6–18); *p* < 0.01]. Tumor size and tumor stage were not associated with the likelihood of undergoing splenectomy (data not shown). In a sensitivity analysis, excluding the laparoscopic procedures did not lead to significant changes in operative time, blood loss, major complication rate, radical resection rate, or survival (data not shown).

## Discussion

This multicenter study on DP for PDAC showed a 33 % major complication rate, a 3 % 30-day mortality, a 6 % 90-day mortality rate, a 5-year cumulative survival of 22 %, and a median survival of 17 months. Independent predictors of survival were an R1/R2 resection, a pT3/pT4 stage tumor, a major complication, and not receiving adjuvant chemotherapy. These predictors can guide future studies aimed at improving survival.

Survival in this series was comparable with a recent multicenter series on DP for PDAC from the US, reporting a median survival of 16 months in 212 patients from nine centers (2000–2008).^10^ Median survival after DP for PDAC in the recent French series was 35 months among 261 patients from 28 centers (2004–2009), surprisingly better than reported elsewhere.^10^,^11^,^23^,^24^ However, time of death was based on hospital records rather than municipal records in that study, and information bias may therefore explain the marked contrast. Although the reported survival could also be related to a different patient selection process or strict adherence to the radical antegrade modular pancreatosplenectomy (RAMPS) technique, details on both aspects are lacking in the report.^11^,^25^ The radical (R0) resection rate in our series was low compared with the literature,^10^,^11^ possibly due to the strict definition for R0 resection, including also a ≥ 1 mm circumferential resection margin. Enhanced adherence to the RAMPS technique could further improve the rate of R0 resection and hence increase survival. In our series, the median number of resected lymph nodes was also comparable with the literature and was not related to average case-volume or the patient’s body mass index.^10^

During the study period, only 45 % of all patients received adjuvant chemotherapy, compared with 69 % in the series from the US and 71 % in the French series.^11^,^23^ This might be due to the early years of the study period, as, in The Netherlands, adjuvant chemotherapy was only considered standard treatment after August 2011. In 2012 and 2013, the amount of patients receiving adjuvant chemotherapy increased to 63 %. Despite the overall higher proportion of patients receiving adjuvant chemotherapy in the series from the US, similar survival outcomes were reported.^10^ As patients who experienced a major complication after DP were less likely to receive adjuvant chemotherapy, it is important to focus on preventing surgical complications. Potentially, a laparoscopic approach could achieve this, although evidence from randomized studies is lacking.^20^ We could not assess the influence of neoadjuvant chemotherapy or radiotherapy on outcomes such as blood loss, resection margins, or mortality, as these treatments were not used within the current study period. Future studies therefore need to determine its impact.

**Fig. 1:**
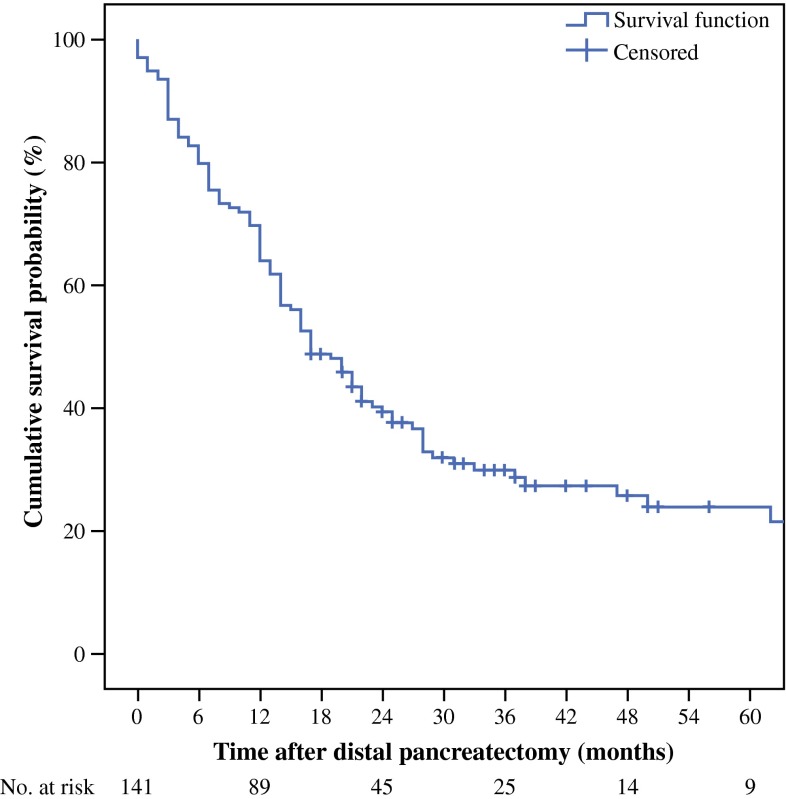
Kaplan–Meier curve of the cumulative survival probability after distal pancreatectomy for pancreatic ductal adenocarcinoma

In the current series, as well as in previously published series, incomplete (R1/R2) resection, pT3/T4 stage, and not receiving adjuvant chemotherapy were significant predictors of worse survival after surgery.^10^,^11^,^23^,^24^,^26^ These results highlight the need for improved surgical technique, patient selection, and attention for adjuvant chemotherapy. Because of the influence of receiving chemotherapy on survival probability, high-quality research on (neo-)adjuvant treatment for patients undergoing surgery because of PDAC is warranted. Only one article described multivisceral resections (21 % of all included DPs) and, in their series, after univariable analysis, multivisceral resection was associated with worse postoperative survival (*p* < 0.01);^11^ however, no quantity of survival worsening, such as median survival or cumulative survival, was given. Therefore, it is unknown how much a multivisceral resection contributes to poor life expectancy (Fig. 1).

In the current series, postoperative morbidity and hospital stay were comparable with reported outcomes in the literature.^27^ This included a 33 % postoperative major complication rate, a 17 % postoperative pancreatic fistula rate, and a median hospital stay of approximately 10 days. The 90-day mortality rate of 6 % is relatively high compared with the literature, but some previous series reported mortality up to 6.3 %.^27^,^28^ Nonetheless, this should be considered the upper limit of acceptable mortality after DP. Given the low number of DPs for PDAC per center in both this and previous series, it could be questioned whether centralization of DPs could improve short-term postoperative outcomes, as has been shown for pancreatoduodenectomy.^29^ Pancreatic surgery has been centralized in The Netherlands as of 2007.^29^ As a consequence, in the first 3 years of this study some patients may have undergone DP in hospitals other than those of the DPCG. However, due to low case-volumes during this period, the total amount of missed patients is expected to be very low.

A limitation of this study is its retrospective design, with the risk of information bias, such as underreporting of study outcomes. Nevertheless, the primary outcome of this study, i.e. survival, is highly reliable since it was recorded using the municipal personal records database.

## Conclusions

Survival after DP for PDAC is poor. Independent predictors of postoperative survival were incomplete tumor resection, advanced tumor stage, major surgical complications, and not receiving adjuvant chemotherapy. This nationwide series on DP for DPAC highlights the importance of preventing surgical complications, improving surgical technique, and the use of adjuvant chemotherapy. Future prospective studies should determine the extent to which these aforementioned measures can improve survival.
